# Built Environment Factors Influencing Walking to School Behaviors: A Comparison between a Small and Large US City

**DOI:** 10.3389/fpubh.2016.00077

**Published:** 2016-04-22

**Authors:** Hyung Jin Kim, Katie M. Heinrich

**Affiliations:** ^1^Landscape Architecture and Regional & Community Planning, Kansas State University, Manhattan, KS, USA; ^2^Kinesiology, Kansas State University, Manhattan, KS, USA

**Keywords:** walking to school, school travel, children, built environment, small city, large city, case-comparison

## Abstract

A growing body of evidence supports the association between the built environment and children walking to school (WTS), but few studies have compared WTS behaviors in cities of different sizes. This case-comparison study utilized WTS data from fourth graders in the small city of Manhattan, KS, USA (*N* = 171, from all eight schools) and data from fourth graders in the large city of Austin, TX, USA (*N* = 671 from 19 stratified-sampled schools). The same survey instrument was used in both locations. After controlling for socioeconomic and demographic variables, built environment, neighborhood, and attitudinal differences were demonstrated by the odds ratios for WTS in the small city vs. the large city. WTS in the small city was more likely to be associated with walking paths/trails and sidewalk landscape buffers en route to school despite lower perceived neighborhood social cohesion, school bus availability, and parental concerns about crime, compared to WTS in the large city. Also, the small city lacked key pedestrian infrastructure elements that were present in the large city. This study highlights important differences related to WTS behaviors and, thus, provides key insights for encouraging WTS in cities of different sizes.

## Introduction

Walking to school (WTS) is a daily routine behavior among school children that can help establish an active lifestyle from early childhood. WTS behaviors add more daily moderate-to-vigorous physical activity than other ways of transporting children to school (1–3). However, studies indicate a nationwide decline in the number of children who are WTS (4, 5).

A growing number of studies have identified personal and socioeconomic factors that contribute to this decline and WTS behaviors in general: gender, age or grade, ethnicity, parent’s socioeconomic status (SES), education level, car ownership, driver’s license, and attitudes and perceptions about WTS (3, 4, 6–16). Also, empirical investigations have shown that environmental factors affect WTS: distance, travel time, safety, urban form and density, land use, and street design (i.e., speed limit, traffic volume, sidewalk, crosswalk, street connectivity) (12, 17–23).

However, such studies have focused primarily on large cities or statewide cross-sectional settings. Rural–urban comparisons of WTS among US children have increased (24–26), but such studies often compared simple attributes like density, population, public transit, or distance, rather than specific characteristics of each setting (4, 27, 28). Such large city-oriented or simple urban–rural classification schemes do not reflect the variety of community settings in the US, ranging from small to large and varying from region to region.

Relatively few studies have considered WTS in smaller US communities, and results from large cities or metropolitan areas may not generalize to smaller cities or towns. In fact, how the built environment supports walking behaviors may differ between small and large cities (29–31). Previous research has considered such correlates of small town walkability as pedestrian infrastructure, land use, proximity to urban services, perceptions, and attitudes, but did not extend their examination to children’s WTS (31, 32). Broader and different regional contexts often affected comparisons of the built environment as it related to walking for adults (33, 34), but few studies consider the similarity of rates or variances of the outcome criteria (e.g., percentage of walking) when determining extent of differences among the compared communities.

This study focused on better characterizing factors in a smaller city that were important for WTS, especially as compared to a larger city. A case-comparison study approach relying on a single analytical instrument was used to compare a small city and a large city in the Central Great Plains region in the US that had similar rates of WTS. This study addressed how different city characteristics influenced WTS behaviors and what may increase the percentage of students’ WTS behaviors in cities of different sizes and with different built environment contexts.

## Materials and Methods

### Setting and Sample

This was a case-comparison study that examined what contributed to children’s WTS behaviors in a small city (case), Manhattan, KS, USA (2010 population: 52,281), and a large city (comparison), Austin, TX, USA (2010 population: 790,390). The total population of Austin was 15.1 times the population of Manhattan and the land area was 15.9 times the area of Manhattan, but their average household size, population densities (persons per square mile), and mean travel times to work were similar. City characteristics are shown in Table 1.

**Table 1:** **Demographic profiles of case-control cities**.

	Small city (case)	Large city (comparison)
	Manhattan, KS, USA	Austin, TX, USA
Total population	52,281 (100.0%)	790,390 (100.0%)
White, non-Hispanic	43,645 (83.5%)	539,760 (63.3%)
Hispanic	3,053 (5.8%)	277,707 (35.1%)
Households	20,008	324,892
Average household size	2.30	2.37
Median age (years)	23.8	31.0
Median household income ($)	61,608	50,520
Land area (square miles)	18.76	297.90
Population density (persons per square mile)	2,786.5	2,653.2
Mean travel time to work (minutes)^a^	15.8	23.2

*^a^Workers age: 16 years+; 2010–2014 data*.

*Sources: 2010 US Census and 2014 American Community Survey (ACS)*.

In 2010–2011, we received 4,602 completed parental surveys (response rates = 33.9%) from 19 stratified-sampled elementary schools out of 74 schools in the Austin Independent School District (AISD); of those responses, 671 fourth grade students were selected as the comparison city group for this study (The total enrollment of AISD fourth grade was 6,673 for that year). In 2013–2014, for Manhattan, the case city, 171 parents of fourth grade students in all eight elementary schools in the Unified School District (USD) 383 completed the same survey (response rates = 41.3%) (the total enrollment of USD 383 fourth grade was 444 for that year). Despite the difference in sample sizes, the observed rates of WTS were similar in both cities: 34.9% in Austin and 28.7% in Manhattan.

### Survey Instrument

The safe routes to school (SRTS) survey instrument were designed based on the National Safe Routes to School survey questionnaire and other related instruments (35–37). This parental survey instrument focused on children’s school travel and behavior outcomes and parental perceptions and attitudes toward children’s physical activity as well as the physical environment on the way to school. The instrument consisted of three groups of questions: (a) school travel factors (i.e., travel mode, environmental features and barriers along the route, sidewalk presence and conditions, walking attitudes and behaviors, safety concerns, travel time, and perceived distance); (b) physical environment-related factors (i.e., neighborhood perceptions, physical barriers, positive and negative environmental changes, and overall walking environmental conditions); and (c) personal and household factors (i.e., children’s gender, grade, race, health, parent’s education, number of siblings, household income, special lunch program, health insurance, pet, family car ownership, etc.). The survey items were constructed after rigorous reliability tests, and the instrument inter-rater reliability was good (Kappa statistics = 0.718 and ICC = 0.998, overall). The City of Austin and Manhattan’s USD 383 helped the research teams by distributing survey questionnaires from school to home. This survey instrument and the study protocol were approved by the IRBs both at Kansas State University and Texas A&M University.

### Procedure

The assessment focused on the differences and similarities between WTS in the small case city and large comparison city. Descriptive statistics and bivariate analyses were performed to identify differences in personal, socioeconomic, attitudinal, and environmental factors between the two settings. First, standard testing procedures were used to identify key variables using unadjusted bivariate analyses and multicollinearity tests among the independent variables. Then, a multiple logistic regression model was conducted to predict the odds of WTS in a small city as compared to large city (reference category); the outcome was a two-category binary variable (WTS in a small city = 1; and WTS in a large city = 0). A model fit statistic (Nagelkerke *R*^2^) was used to develop an optimal multivariate model, and a significance level of 0.05 was used as a threshold to determine statistical significance. SPSS 20.0 (IBM Corp., Armonk, NY, USA) was used for data analysis.

## Results

### Differences in Personal and School-Neighborhood Characteristics for the Small City vs. the Large City

#### Socioeconomic Status

As shown in Table 2, children from the small city were more likely to be White (69.7 vs. 23.9%), have more cars per household (2.1 cars vs. 1.5 cars), have parents with higher education degrees (college or higher degree: 91.8 vs. 44.5%), with fewer qualified for free or reduced school lunch program (32.0 vs. 66.2%) than children from the large city. Overall, fourth grade school children and their parents in the small city could be characterized as having higher SES and education levels with more White residents than those from the large city (Table 2).

**Table 2:** **Descriptive statistics and bivariate analyses for a small city and a large city: SES, school travel, and neighborhood characteristics**.

Variable		Descriptive statistics				Bivariate analyses	
		Small city		Large city			
		*n*	% or mean (SD)	*n*	% or mean (SD)	*t* or χ^2^	Sig.
Child’s race	Non-Hispanic White (%)	165	**69.7%**	641	23.9%	124.172	<0.001
Special lunch program	Child qualified for special school lunch programs (free or reduced price, %)	169	32.0%	598	**66.2%**	63.810	<0.001
Car ownership	How many cars are there in your household? (# of cars per household)	171	**2.06** (0.87)	631	1.52 (0.78)	−7.450	<0.001
Education	Household’s highest education (college or higher degree, %)	171	**91.8%**	646	44.5%	121.618	<0.001

BMI	Child’s body mass index (BMI)	134	17.98 (3.45)	394	**21.48** (8.94)	6.483	<0.001
WTS	Walking to/from school on a normal week day (%)	171	28.7%	671	34.9%	2.361	0.124
Perceived distance	Is this distance close enough for your child to walk to school? (yes, %)	171	53.8%	635	54.8%	0.055	0.815
School travel time	How long does it take to get to school? (minutes)	166	9.29 (6.40)	618	**11.13** (9.72)	3.068	0.002
School bus availability	Does the school provide bus service for your child? (yes, %)	169	**40.2%**	652	30.1%	6.370	0.012
School volunteer	Have you volunteered at your child’s school in the past 12 months? (yes, %)	171	**51.5%**	647	39.4%	8.065	0.005

Reasons for neighborhood choice	Housing/rent price (%)	171	**48.0%**	655	35.4%	9.040	0.003
	Close to my child’s school (%)	171	32.7%	655	**46.1%**	9.854	0.002
	Quality of neighborhood (%)	171	**52.0%**	655	34.8%	17.037	< 0.001

*Bivariate analyses include t-test and Chi-square test results; and sig. is p-value*.

*Significantly higher values are noted in bold*.

#### BMI and School Travel

Although children from the large city had higher BMIs than children from the small city (21.48 vs. 17.98), differences in the percentage of children WTS were insignificant (34.9% for the large city vs. 28.7% for the small city; *p* = 0.124). More than 50% of parents for both groups reported that school was close enough for their children to walk, with no significant differences (54.8% for the large city vs. 53.8% for the small city; *p* = 0.816), but the average travel time was less by 1 min 50 s for children in the small city (9.29 min as compared to 11.13 min). Small city schools provided more school bus service (40.2 vs. 30.1%), and more parents in the small city than the large city were involved with school (school volunteering in the last year: 51.1 vs. 39.4%) (Table 2).

#### Neighborhood Choice

As shown in Table 2, proximity to the child’s school mattered more to parents in the large city than the small city (46.1 vs. 32.7%), while housing/rent prices and neighborhood quality were more important to the small city parents.

#### Parental Safety Concern

Parental concerns about the safety of WTS were rated from 1 (Strongly disagree) to 5 (Strongly agree). Significantly greater concerns for small city parents included their child being bullied, teased, or harassed when WTS and being hit by a car. Large city parents expressed significantly more concern about stray dogs, exhaust fumes, and lack of people seeing and helping their child in case of danger (Table 3).

**Table 3:** **Descriptive statistics and bivariate analyses for a small city and a large city: parental safety concerns**.

	Variable	Descriptive statistics				Bivariate analyses	
		Small city		Large city		*t*	Sig.
		*n*	Mean (SD)	*n*	Mean (SD)		
Safety concern about WTS (Likert scale)	My child may get bullied, teased, or harassed	168	**3.20** (1.20)	646	2.65 (1.51)	−5.025	<0.001
	My child may be attacked by stray dogs	169	3.25 (1.21)	647	**3.64** (1.37)	3.628	<0.001
	My child may be hit by a car	167	**3.92** (1.17)	647	3.23 (1.40)	−6.528	<0.001
	Exhaust fumes may harm my child’s health.	169	2.47 (1.17)	645	**3.28** (1.44)	7.563	<0.001
	No one will be able to see and help my child in case of danger	169	3.21 (1.27)	645	**3.72** (1.39)	4.369	<0.001

*Bivariate analyses include *t*-test results; Sig. is *p*-value; and for Likert scale, 1 = lowest (strongly disagree) and 5 = highest (strongly agree) rating*.

*Significantly higher values are noted in bold*.

### Differences in Environmental Characteristics En Route to School for the Small City vs. the Large City

**Figure 1:**
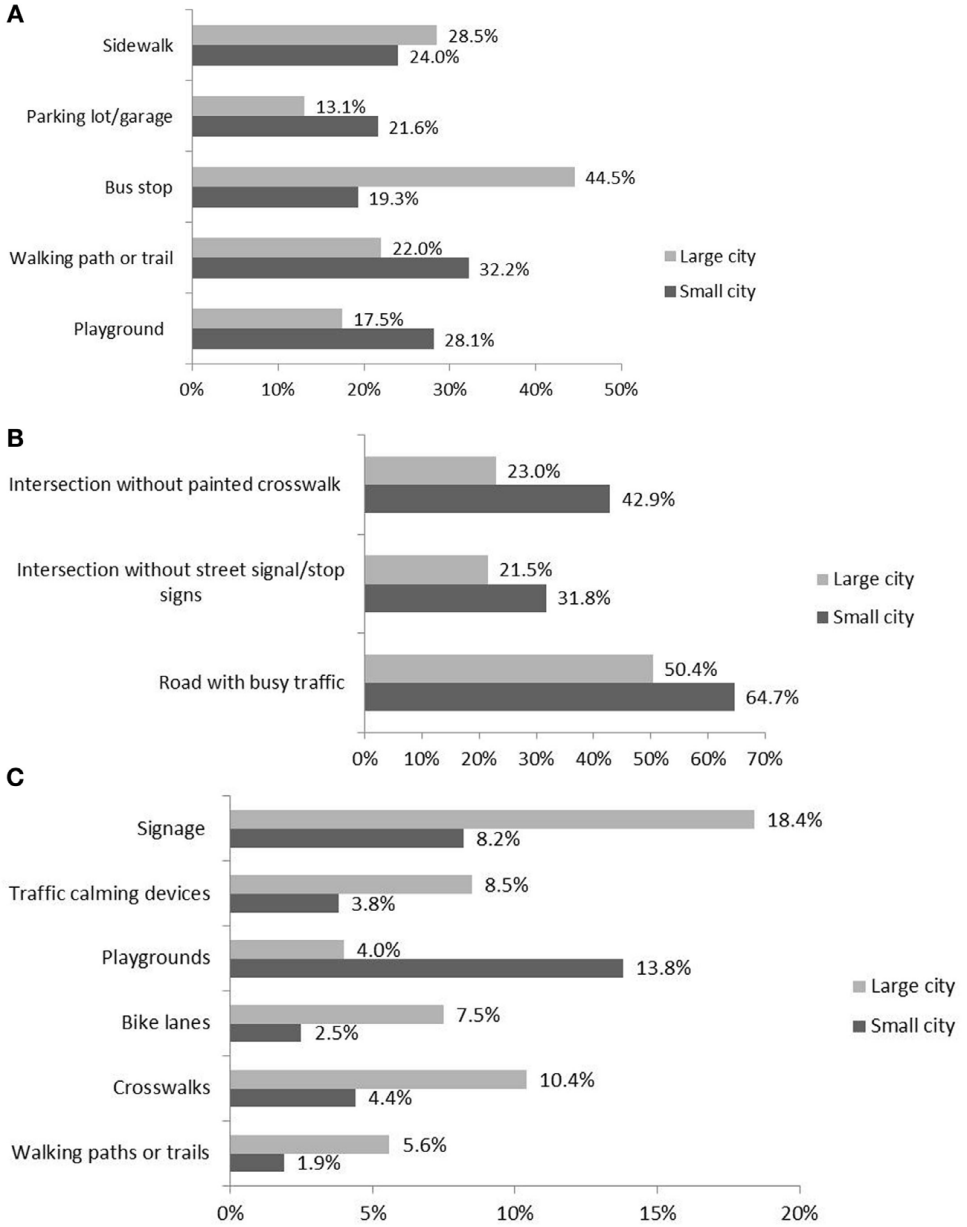
**Built environmental characteristics en route to school for a small city and a large city**. Note: only the significant results from chi-square tests are reported (*p* < 0.05). **(A)** Environmental features en route to school, **(B)** crossing barriers en route to school, and **(C)** environmental changes en route to school (in the past year).

When traveling along the route to school, small city children were less likely to have sidewalks (24.0 vs. 28.5%) and bus stops (19.3 vs. 44.5%), but they had more parking lots/garages (21.6 vs. 13.1%), walking paths or trails (32.2 vs. 22.0%), and playgrounds (28.1 vs. 17.5%) than children in the large city. However, small city children crossed more barriers like intersections without painted crosswalks (42.9 vs. 23.0%) or street signals/stop signs (31.8 vs. 21.5%) as well as crossing more heavily traveled roads (64.7 vs. 50.4%) than large city children. Also, large city children traveled routes to school with more recent infrastructure improvements like signage (e.g., school zone, child crossing warning) (18.4 vs. 8.2%), traffic calming devices (8.5 vs. 3.8%), bike lanes (7.5 vs. 2.5%), crosswalks (10.4 vs. 4.4%), or walking paths/trails (5.6 vs. 1.9%), while small city children saw more playground changes (13.8 vs. 4.0%). Overall, large city children had exposure to more pedestrian infrastructure improvements en route to school than small city children. See Figure 1 for more details.

### Results of Multivariate Analyses

After adjusting for personal demographic and socioeconomic variables, a multiple logistic regression model was estimated to predict the odds of WTS in Manhattan, the small city, compared to Austin, the large city (dependent variable: WTS in the small city = 1 and WTS in the large city = 0) (Table 4). Among 283 children who walked to school (33.6% of the whole sample from both cities), 49 walked to school in the small city (28.7% of the Manhattan sample) and 234 walked to school in the large city (34.87% of the Austin sample). The Nagelkerke *R*^2^ of the final model was 0.869 (Cox and Snell *R*^2^ was 0.577). Only the significant results (*p*-value <0.05) are reported in the text below.

**Table 4:** **Multiple logistic regression model: variables predicting the odds of WTS in a small city vs. in a large city**.

Independent variable	OR	Sig.	95% CI	
			Lower	Upper
**Base model (individual and household SES)**				
Child race (1 = White; 0 = other)	540.68	0.001	14.49	20169.34
Household car ownership (number of cars)	22.24	0.004	2.68	184.40
School lunch program (1 = reduced/free lunch; 0 = none)	7.22	0.172	0.42	122.96
Highest education level of household (1 = college or higher; 0 = lower than college)	38.76	0.009	2.51	598.81

**Built environment**				
Walking path or trail en route to school (1 = present; 0 = none)	12.51	0.026	1.36	115.46
Sidewalk landscape buffer (Separated from traffic by grass/trees) (1 = present; 0 = none)	4.80	0.000	1.99	11.58
School bus availability (1 = available; 0 = none)	0.01	0.028	0.00	0.63

**Safety concern**				
Traffic safety^a^ (“My child may be hit by a car.”)	14.43	0.002	2.74	76.01
Air pollution^a^ (“Exhaust fumes may harm my child’s health”)	0.41	0.053	0.17	1.01
Crime surveillance^a^ (“No one will be able to see and help my child in case of danger.”)	0.22	0.006	0.07	0.65

**Social environment**				
Social cohesion^a^ (“I feel connected to people in my neighborhood.”)	0.44	0.044	0.20	0.98

*Reference category is “WTS (walking to school) in the large city*.”

*^a^5-point Likert scale with 1 = (strongly disagree) and 5 = highest (strongly agree) ratings*.

*Sig. is *p*-value; OR is odd ratio; and CI is confidence interval*.

#### Base Model (Individual and Household SES)

Individual- and household-level SES variables were constructed as a base model using a theoretical foundation from empirical studies of WTS through one-by-one bivariate analyses. In this study, child’s ethnicity (White vs. non-White), participation in a school lunch program (reduced or free lunch program; not significant in the final model), household’s car ownership (# of cars), and highest education level (college or higher vs. less than college) were selected for predicting the estimated odds ratio for WTS in the small city vs. the large city. The Nagelkerke *R*^2^ of the base model was 0.488, which represented 56.6% of the variance of the final model. From the final model results, the households of children who WTS in the small city were more likely to be White (OR = 540.68, 95% CI = 14.49–20,169.34), own more cars (OR = 22.24; 95% CI = 2.68–184.40), and have college or higher degrees (OR = 38.76; 95% CI = 2.51–598.81).

#### Built Environment

Walking to school in the small city meant children were more likely to encounter informal walking paths or trails en route to school than in the large city (OR = 12.51; 95% CI = 1.36–115.46). This indicated that small city schools might be in more natural or less-designed neighborhood environments. Also, parents of small city walkers were more likely to report landscape buffers (grass or trees) between the sidewalk and the road en route to school than parents of large city walkers (OR = 4.80; 95% CI = 1.99–11.58). The availability of school bus service was not a direct environmental factor but was critical for school travel choice and related to home-to-school distance. In the final model, WTS in the small city was less likely to be associated with school bus availability than the large city (OR = 0.01; 95% CI = 0.00–0.63), although the small city had better availability of school bus service than the large city (Table 2).

#### Other Correlates

Three parental safety concern variables contributed to the final model. Parents in the small city who allowed their children to WTS were more concerned about traffic safety (“My child may be hit by a car”; OR = 14.43; 95% CI = 2.74–76.01) but were less worried about crime (“No one will be able to see and help my child in case of danger”; OR = 0.22; 95% CI = 0.07–0.65) than parents in the large city. Also, in the small city’s social environment, WTS was less likely to be associated with social cohesion than the large city (“I feel connected to people in my neighborhood”; OR = 0.44; 95% CI = 0.20–0.98).

## Discussion

### Findings

In this study, we examined what affects the choice to WTS in a small city in contrast to a large city through case-comparison. The population and area of the large city, Austin, were higher than the small city, Manhattan, but their population densities were similar, indicating the small city had a compact urban pattern similarly as the large city (38). Although the two cities had similar rates of WTS behaviors reported by fourth grade parents, key differences were found for the factors related to WTS, particularly perceived home-to-school distance and proximity to school, which were less important determinants in choosing a neighborhood in the small city. Moreover, in this study, the mean BMI among children was lower in the small city than in the large city. This result can be directly compared to other studies that have noted the prevalence of higher childhood obesity in smaller towns in rural areas than in urban areas (39, 40).

Bivariate analysis results revealed both similarities and differences in what affected WTS in both cities. The small city had shorter home-to-school travel time, more school bus availability, and more parental school volunteering. Children from the small city were less benefited by pedestrian infrastructure improvements and exposed to more barriers to WTS than children in the large city. Our results indicated the overall lack of pedestrian infrastructure and its improvement in the small city, while the large city had both pedestrian infrastructure and fewer barriers to walking. Accordingly, rates of WTS were 6.2% higher in the large city, but this difference was not statistically significant. Comprehensive supports can be important for ensuring continuous quality improvement of pedestrian environments in many small US cities, such as the Safe Routes to School program. However, the Safe Routes to School program recommends matching engineering solutions (i.e., pedestrian environment improvements) to the type of problem presented by each community and those solutions need to address both the infrastructure at schools and that along a child’s route to school (41).

Furthermore, different factors predicted WTS in the small city than the larger city. The base model variables were consistent with previous studies: WTS in the small city correlated more with race (White or not), household car ownership, and parents’ highest education level than in the large city (3, 4, 9, 42). After controlling for base model variables, walking paths/trails and sidewalk landscape buffers (grass/trees) were positive built environment correlates of WTS in the small city. Trails, as a correlate, were an interesting finding. The existence of additional travel paths can shorten the travel distance between home and school (43). Further studies need to target recreational walking or behaviors in and around walking trails, particularly in small cities. Street design policies and guidelines that can incorporate existing trails and accommodate pedestrian travel may be important in promoting WTS as much in smaller cities as large cities.

Previous research has found that neighborhood social cohesion can positively influence physical activity behaviors for youth (44). Compared to the small city, social cohesion and school bus availability contributed more to WTS in the large city. School policy often dictates the minimum distance from school that children must live to be eligible to ride the bus. In the small city of Manhattan, KS, USA, students must either live 2.5 miles away from their school or in a designated Hazard area (45). In the large city of Austin, TX, USA, the minimum residence distance from school for school bus eligibility is 2 miles, measured along the shortest route to school (46). In addition, the Austin policy states that children who live closer than 2 miles may be eligible for bus service if they are “subject to hazardous traffic conditions if they were to walk to school.”

Parental attitudes and concerns about safety in WTS were significantly different for both cities, but in particular, in the small city, Manhattan, concerns about children being hit by cars was an immediate barrier to WTS. Similar research in urban and rural schools in Hawaii found that both the speed and amount of traffic, along with safety of intersections/crossings were key factors influencing parents’ decisions regarding children WTS (47). Projects in California to improve traffic safety along routes to school (e.g., installation/widening of crosswalks and sidewalk improvements) resulted in significantly greater walking or biking to school for children who encountered the improvements along their usual route (48). The small city of Manhattan, KS, USA has applied for SRTS funding to help construct such safety improvements around the city schools, and future research can help determine their effect on changes in WTS.

The slightly higher population density in the small city of Manhattan, KS, USA reflects the city’s emphasis on having a compact development pattern encouraging growth within the Urban Service Area Boundary with emphasis on infill and redevelopment (49). Most Manhattan elementary schools are located in residential areas, with many students residing within walking distance. By contrast, the large city Austin, TX, USA comprehensive plan acknowledges that urban sprawl is a problem, with land area growth of over 19% between 2000 and 2010 (50).

### Limitations and Conclusion

Several limitations of this study need to be noted. First, in using a comparative approach with purposefully selected samples, the results are not generalizable to every small and large city in the US. Our intention was to provide valuable and comparable insights into an understudied comparison between communities. Second, the use of data from two studies might have limited the analysis, but a single instrument and analytic approach were used as a way to reduce potential analytical errors. Third, statistical power in comparison may be weak, and some variables may not be comparable because sample sizes were unequal or small or samples may have had different sociodemographic characteristics. Fourth, the 2- to 3-year time difference in data collection may have weakened the results and limit applicability to current practices and policies.

Some additional limitations were also present. Not all confounding potential variables were considered in the final model. Self-reported data, such as percentage of WTS, BMIs, or parental perceptions, may cause misclassification or overstatement of categories. Despite these limitations, this study has added to the current literature on WTS, highlighting important differences related to WTS in a small city as opposed to a large one and, thus, providing an opportunity to offer some policy and design implications to help smaller cities encourage children to walk to school.

In summary, this study compared the effects of built environment and other factors on whether children walk to school in a small or large city, each with individual characteristics. Different correlates of the built environment and socioeconomic and attitudinal aspects were associated with WTS in each city. Our findings suggest that high-quality pedestrian infrastructure can encourage WTS in a small city, as well as integrating green infrastructure with traditional infrastructure at street level, widening sidewalks, adding a landscape buffer, or using natural walking paths/trails. The relative lack of pedestrian infrastructure in smaller cities should be further researched. In addition, comparable cases at multiple community settings should provide further evidence for how WTS can be encouraged. We recommend researching more diverse community samples to avoid the urban–rural dichotomy.

## Author Contributions

HK conceived of this study, performed data analysis, drafted manuscript, and assumed the responsibility for the manuscript; and KH prepared the manuscript and provided conceptual advice. All authors made significant contributions to this research.

## Conflict of Interest Statement

The authors declare that the research was conducted in the absence of any commercial or financial relationships that could be construed as a potential conflict of interest.
